# Breast Arterial Calcifications on Mammography among Patients Attending the Radiology Department in a Tertiary Care Centre: A Descriptive Cross-sectional Study

**DOI:** 10.31729/jnma.6922

**Published:** 2021-09-30

**Authors:** Anamika Jha, Anugya Sah, Birendra Raj Joshi, Benu Lohani

**Affiliations:** 1 Department of Radiology, Tribhuvan University Teaching Hospital, Maharajgunj Medical Campus, Maharajgunj, Kathmandu, Nepal

**Keywords:** *atherosclerosis*, *calcification*, *mammography*

## Abstract

**Introduction:**

Breast arterial calcifications are common mammographic findings which are associated with coronary artery disease. The aim of this study was to find the prevalence of breast arterial calcifications in women presenting for mammography in a tertiary care centre.

**Methods:**

This descriptive cross-sectional study was performed in the Department of Radiology, in a tertiary care hospital after taking ethical clearance, Reference number 352(6-11)E-2, 077/078, data was collected from Syngovia database from March-June 2021 which included 1614 mammograms. Convenience sampling was done and mammograms evaluated for presence of vascular or non-vascular calcification. Further, vascular calcification was graded. Data was entered in Statistical Package for Social Sciences version 25. Point estimate at 95% Confidence Interval was done, and frequency and proportion were calculated.

**Results:**

The prevalence of breast arterial calcification was 188 (11.6%) at 95% Confidence Interval (10.03-13.2). The mean age of women included in this study was 48.42±9.55 years with the largest number of patients in the age group 40-49 years, 682 (42.3%), and least in the age group 80-89 years, 3 (0.2%). All patients in the age group 80-89 years, 3 (100%) had vascular calcifications followed by 70-79 years group, 22 (57.5%) and none in patients younger than 30 years.

**Conclusions:**

We found an increase in the number and grade of vascular calcifications in breasts with the patient's age. When present breast arterial calcifications must be mentioned in mammogram report. Identification of such calcifications on mammogram should prompt further screening for atherosclerotic disease.

## INTRODUCTION

Mammograms are commonly done for screening of breast cancer or evaluation of breast symptoms. In Nepal, diagnostic mammograms are done more often than screening due to lack of awareness and absence of guidelines facilitating screening studies.^1^ Breast arterial calcifications (BAC) represent arteriosclerotic changes occurring in medium sized arteries supplying the breast.

Different calcifications, including vascular, are often seen in mammograms, but being unrelated to breast disease, are often not reported. Various studies have shown an increased risk of Coronary Artery Disease (CAD) with BAC.^2-4^ In addition to CAD, other risk factors like age, Diabetes Mellitus, CAD risk factors, pregnancy, lactation and renal impairment are also associated with BAC.^5^ Reporting BAC may help identify group of patients at higher risk of CAD and prompt appropriate and timely evaluation.

The aim of our study was to find the prevalence of BAC in women presenting for mammography in our tertiary care hospital.

## METHODS

This was a descriptive cross-sectional study conducted in the Department of Radiology, Tribhuvan University Teaching Hospital (TUTH). Data was collected from the database over a period of 4 months (March-June 2021). Ethical clearance [Reference number 352(6-11)E-2, 077/078] was obtained from the Institutional Review Committee and patient confidentiality was maintained. Convenient sampling technique was used. Patients with breast implants or previous breast surgery deforming the breast, rendering evaluation difficult were excluded.

The sample size was calculated using the formula,

n = Z^2^ × p × q / e^2^

  = (1.96)^2^ × 0.5 × 0.5 / (0.03)^2^

  = 1,067

where,

n = required sample size
p = prevalence 50%
q = 1-p
e = margin of error, 3%
Z = 1.96 at 95% Confidence Interval (CI)

Adding a non-response rate of 10%, the sample size was 1,174. However, 1614 samples were included in the study.

Two basic projections (cranio-caudal & mediolateral oblique) of the breasts were obtained with the MAMMOMAT Fusion mammographic unit. These mammograms available in Syngovia work station YLXR019095 database were evaluated by radiologists with more than 10 years of expertise. The pattern and extent of calcification was evaluated visually in the workstation using appropriate magnification tools. Presence of calcification of any type was noted and classified as benign vascular, benign non-vascular or malignant. Vascular calcification was further graded as follows, which was adapted from the previously proposed scoring systems.^6,7^

0. No vascular calcification;
1. Few punctate vascular calcifications with no coarse, tram track or ring calcifications;
2. Abundant punctate vascular calcification or tram track calcifications;
3. Severe coarse or tram track calcification obliterating or clouding the lumen.

Study variables were recorded in a predesigned datasheet. Statistical analyses were done with the SPSS version 25.0 and Microsoft Excel. Simple statistical measures were used to determine the overall prevalence and grade of calcification and that in the different age groups.

This study was done as a part of the University Grant Commission project FRG-HS-3 075-76.

## RESULTS

The prevalence of BAC in our study was found to be 188 (11.6%) at 95% CI (10.03-13.2).

**Figure 1:**
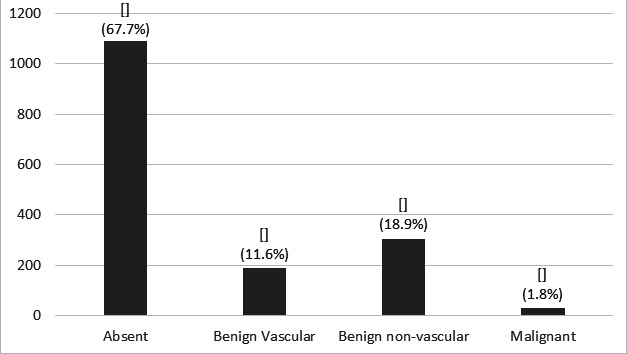
Overall distribution of different types of calcifications in mammogram.

The study sample consisted of 1614 female patients with age ranging from 20 to 89 years, mean age of 48.42±9.55 years. The maximum number of patients were in 40-49 years, 682 (42.3%) age group and minimum in 80-89 years, 3 (0.2%) (Table 1, Figure 1).

**Table 1:** Distribution of different types of calcifications in various age groups.

Age group	Calcification Absent	Benign Vascular	Benign non-vascular	Malignant	Total number in various age groups.
20-29	21 (1.3)	0 (0)	1 (0.06)	0 (0)	22 (1.4)
30-39	171 (10.5)	7 (4.3)	27 (1.7)	8 (0.5)	213 (13.2)
40-49	524 (32.4)	29 (1.8)	118 (7.3)	11 (6.8)	682 (42.3)
50-59	293 (18.1)	57 (3.5)	112 (6.9)	7 (0.4)	469 (29.1)
60-69	74 (4.5)	70 (4.3)	39 (2.4)	2 (0.1)	185 (11.5)
70-79	9 (0.5)	22 (1.4)	8 (0.5)	1 (0.06)	40 (2.5)
80-89	0 (0)	3 (0.2)	0 (0)	0 (0)	3 (0.2)
Total	1092 (67.6)	188 (11.6)	305 (18.8)	29 (1.8)	1614

The mean age of patients without vascular calcification was 46.32±8.38 and that with vascular calcification was 58.13±10.27 years. A total of 22 (1.4%) women in the age group 20-29 years and 213 (13.2%) women in the age group 30-39 years in our study were below the recommended age for mammography, which is suggested for women above the age of 40. However, these women had risk factors like personal or family history of breast cancer, or had undergone diagnostic mammography.

**Figure 2:**
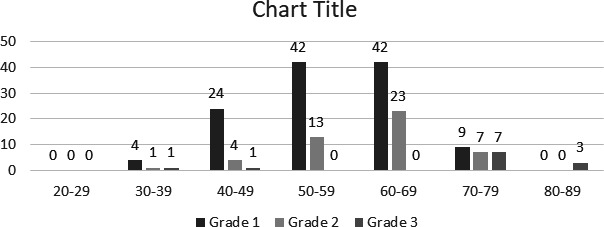
Bar diagram of the distribution of grades of BAC according to age group.

Calcifications were seen in a total of 522 (32.3%) mammograms of which 188 (36%) were benign vascular, 305 (58.4%) were benign non vascular and 29 (5.5%) malignant (Figure 2 and Table 1). The prevalence of BAC in our study was 188 (11.6%) with a maximum number in the 60-69 years age group. There were 121 (7.5%) cases with grade 1, 48 (3%) with grade 2 and 19 (1.2%) with grade 3 calcification. Maximum percentage of grade 1 calcification was in 50-59, grade 2 in 60-69 and grade 3 in 80-89 years age groups (Figure 2).

**Figure 3:**
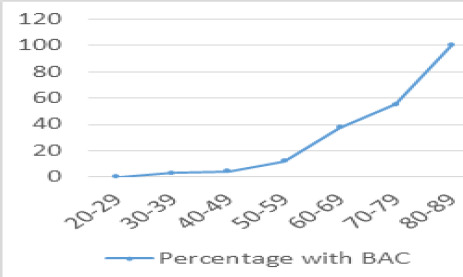
Percentage distribution of BAC with age.

There was an increase in prevalence of BAC with increasing age, with no calcifications in less than 29 years of age and all patients older than 80 years having BAC (Figure 3).We found increase in the number and grade of BAC with age.

## DISCUSSION

BAC more often involves calcification in the tunica media and represents arteriosclerosis than atherosclerotic intimal calcification. These are fine initially, becoming a more definite linear or tram track followed by dense, coarse and confluent obscuring the vessel lumen.^8^ It may be difficult to differentiate medial or intimal calcification on mammogram. Studies have shown a higher incidence of BAC in patients with renal disease, atherosclerotic coronary artery disease, CAD risk factors, diabetes as well as previous history of pregnancy and lactation.^5,9,10^ McLenachan, et al. concluded that while absence of BAC has a high negative predictive value for ruling out CAD, the diagnostic accuracy to predict the same is limited.^11^ Some other studies do not show any association between BAC and CAD.

We had more patients with grade 1 calcification than grade 2 or 3 and an increasing number and grade of BAC with age, with all those above 80 years of age having grade 3 BAC. The increase in presence and grade of BAC with age in our study is consistent with most other studies.

The prevalence of breast calcifications in our study of any type, that is, vascular or non'vascular was about 522 (32.3%) and BAC was 188 (11.6%). Studies have shown the prevalence of BAC generally varies from 9-17%, being higher in older patients.^3^ A similar study which had population ranging from 49-70 years, showed prevalence similar to our study of 11%, and found a positive association with age, pregnancy and lactation. However, they did not find any relation between BAC and atherosclerotic risk factors.^12^ The prevalence of BAC was 14.1% in another study, including patients from 40-93 years of age, with increasing trend with age.^2^ Higher prevalence of 20% was seen in a study conducted in Nigeria.^3^ Reddy J, et al. found the overall prevalence of BAC to be 29.4% in patients from 35-92 years of age, which varied with ethnicity, being 34.5% in Hispanics, 24% in whites and 7.1% for Asians.^13^ Compared to our study, while most others have greater BAC prevalence, some show a lower prevalence, minimum being 3%. The slightly lower prevalence in our study could be due to a greater number of younger patients with diagnostic indications more often than screening mammograms.^1^ We had a few patients younger than 40 years, who either had high risk factors for breast carcinoma or another diagnostic indication.

While such vascular calcifications are often seen on mammography, being unrelated to breast cancer, they are not always mentioned in the report. An interesting study found that patients and preventive cardiologists prefer BAC being reported in mammogram studies done for other indications.^14^ It is important to make a note on this incidental finding.^15^

## CONCLUSIONS

Since there is an increase in prevalence and grade of vascular calcification with age. We recommend that the benign vascular calcification when present must always be reported in the mammogram report, as such patients may have higher risk of atherosclerotic disease.

## References

[1] Giri M, Giri M, Thapa RJ, Upreti B, Pariyar B (2018). Breast Cancer in Nepal: Current status and future directions.. Biomedical Rep..

[2] Kelly BS, Scanl ON E, Heneghan H, Redmond CE, Healy GM, Mc Dermott E, Heffernan EJ, Prichard R, Mc Nally S (2018). Breast Arterial Calcification on screening mammography can predict significant Coronary Artery Disease in women.. Clin Imaging..

[3] Akinola RA, Ogbera OA (2011). Mammograms and breast arterial calcifications: looking beyond breast cancer: a preliminary report.. BMC Res Notes.

[4] Moshyedi AC, Puthawala AH, Kurland RJ, O'Leary DH (1995). Breast arterial calcification: Association with coronary artery disease. Work in progress.. Radiology..

[5] Ali E.A., Fouad H., Razek N.A. (2019). Evaluation of mammography detected breast arterial calcifications as a predictor of coronary cardiac risk.. Egypt J Radiol Nucl Med.

[6] Loberant N, Salamon V, Carmi N, Chernihovsky A (2013). Prevalence and Degree of Breast Arterial Calcifications on Mammography: A Cross-sectional Analysis.. J Clin Imaging Sci..

[7] Mostafavi L, Marfori W, Arellano C, Tognolini A, Speier W, Adibi A (2015). Prevalence of coronary artery disease evaluated by coronary CT angiography in women with mammographically detected breast arterial calcifications.. PloS one..

[8] Kim H, Greenberg JS, Javitt MC (1999). Breast calcifications due to Monckeberg medial calcific sclerosis.. Radiographics..

[9] Pecchi A, Rossi R, Coppi F, Ligabue G, Modena MG, Romagnoli R (2003). Association of breast arterial calcifications detected by mammography and coronary artery calcifications quantified by multislice CT in a population of post-menopausal women.. Radiol Med..

[10] Rotter MA, Schnatz PF, Currier AA, O'Sullivan DM (2008). Breast arterial calcifications (BACs) found on screening mammography and their association with cardiovascular disease.. Menopause..

[11] McLenachan S., Camilleri F., Smith M., Newby D.E., Williams M.C. (2019). Breast arterial calcification on mammography and risk of coronary artery disease: a SCOT-HEART sub-study.. Clin Radiol.

[12] Maas AH, van der Schouw YT, Beijerinck D, Deurenberg JJ, Mali WP, van der Graaf Y (2006). Arterial calcifications seen on mammograms: cardiovascular risk factors, pregnancy, and lactation.. Radiology..

[13] Reddy J, Son H, Smith SJ, Paultre F, Mosca L (2005). Prevalence of breast arterial calcifications in an ethnically diverse population of women.. Ann Epidemiol..

[14] Margolies LR, Yip R, Hwang E, Oudsema RH, Subramaniam VR, Hecht H, Narula J (2019). Breast Arterial Calcification in the Mammogram Report: The Patient Perspective.. Am J Roentgenology.

[15] Fiuza Ferreira EM, Szeinfield J (2007). Faintuch S: Correlation between intramammary arterial calcifications and CAD.. Acta Radiol..

